# Assessing the annual burden of tick-borne encephalitis virus infections, north-east Italy, 2017 to 2024

**DOI:** 10.2807/1560-7917.ES.2026.31.17.2500733

**Published:** 2026-04-30

**Authors:** Emmanouil Alexandros Fotakis, Martina Del Manso, Piero Poletti, Giorgio Guzzetta, Antonino Bella, Chiara Sacco, Debora Ballarin, Cristina Zappetti, Maria Grazia Zuccali, Silvia Spertini, Flavia Riccardo, Patrizio Pezzotti, Stefano Merler, Tick-borne encephalitis group, Tick-borne encephalitis group, Filippo da Re, Francesca Zanella, Davide Gentili, Sarah Samez, Cristina Schellenberger, Chiara Mocellin, Elisabetta Pagani

**Affiliations:** 1 Center for Health Emergencies, Bruno Kessler Foundation, Trento, Italy; 2 Department of Infectious Diseases, Istituto Superiore di Sanità, Rome, Italy; 3 Directorate of Prevention, Food Safety, and Veterinary Public Health-Veneto Region, Venice, Italy; 4 Central Directorate for Health, Social Policies, Friuli Venezia Giulia Regione, Udine, Italy; 5 Department of Prevention, Provincial Health Authority, Trento, Italy; 6 Public Health and Hygiene Service, South Tyrol, Italy; 7 External Relations Office and Centre for International Affairs, Istituto Superiore di Sanità, Rome, Italy; 8 The members of the Tick-borne encephalitis group are listed under Collaborators

**Keywords:** Italy, Triveneto, Tick borne encephalitis, burden, DALYs

## Abstract

**BACKGROUND:**

Tick-borne encephalitis (TBE), a neuroinvasive disease in humans, is endemic in north-east Italy and nationally notifiable since 2017. Domestic TBE incidence, although low, has increased in recent years while TBE vaccination coverage remains < 10%.

**AIM:**

We aimed to estimate the burden of TBE virus (TBEV) infections in north-east Italy (Triveneto) in 2017–2024.

**METHODS:**

We estimated disability-adjusted life years (DALYs) with 95% uncertainty intervals (UI) using the Burden of Communicable Diseases in Europe software. Input data included TBE cases notified in 2017–2024 with residence and/or infection or exposure in Triveneto (autonomous provinces (AP) of Trento and Bolzano and regions of Veneto and Friuli-Venezia Giulia) and correction factors for TBE underdiagnosis and TBEV infection under-ascertainment.

**RESULTS:**

In 2017–2024, 295 TBE cases were notified in Triveneto. The mean annual burden of TBEV infections was estimated at 0.58 (95% UI: 0.55–0.62) DALYs per 100,000 population. Estimates were highest in 2022 (1.07; 95% UI: 1.00–1.14 DALYs/100,000). The areas with the highest burden were Belluno province (4.17; 95% UI: 3.88–4.46 DALYs/100,000/year) and the AP of Trento (2.84; 95% UI: 2.65–3.05 DALYs/100,000/year). Males accounted for 67.0% of DALYs. All age groups experienced on average ≤ 1 DALYs per 100,000 population per year.

**CONCLUSION:**

The burden of TBEV infections in north-east Italy is relatively low and with an uneven geographical distribution. Increasing TBE vaccination coverage across age groups, prioritising elevated impact areas, may reduce the burden of TBEV infections in north-east Italy and maintain TBE as a low-burden disease.

**Key public health message**

**What did you want to address in this study and why?**

Tick-borne encephalitis (TBE) can be a severe disease affecting the central nervous system. We wanted to quantify the health burden (i.e. deaths and disability) caused by TBE virus (TBEV) infections in north-east Italy, in 2017-2024.

**What have we learnt from this study?**

The annual burden of TBEV infections in north-east Italy in 2017–2024 was low, 0.58 disability-adjusted life years (DALYs) per 100,000 population, yet in recent years it exceeded the European average of 0.69 DALYs per 100,000 population per year estimated for 2009–2013. Most of the burden is attributed to chronic disability. Within north-east Italy, burden estimations differed between areas and by sex, with males experiencing approximately two thirds of the total burden.

**What are the implications of your findings for public health?**

This study identified a non-trivial health burden attributed to TBEV infections in north-east Italy, requiring public health attention. Our estimates indicate the need of ensuring high TBE vaccination coverage across all age groups in local populations to maintain the low burden of TBE.

## Introduction

Tick-borne encephalitis (TBE) is a disease of the central nervous system (CNS) caused by tick-borne encephalitis virus (TBEV, *Orthoflavivirus* genus, Flaviviridae family) which is transmitted to humans predominantly by bites of infected ticks [1]. Transmission may also occur via consumption of contaminated unpasteurised dairy products [2].

Humans are considered as dead-end hosts. Over 70% of TBEV infections in humans caused by the European virus subtype, the predominant virus subtype in the European Union/European Economic Area (EU/EEA), are asymptomatic [3]. Central nervous system involvement is estimated to occur in 20–30% of symptomatic infections [4], although the proportion of patients with CNS symptoms varies [5]. Tick-borne encephalitis attributed to the European TBEV subtype typically manifests as a biphasic disease. The first phase begins with an unspecific febrile illness. The second phase involves the CNS with signs of meningitis, encephalitis, myelitis or radiculitis [6]. Case fatality rate of neuroinvasive cases is low (< 2%), but TBE can cause long-lasting morbidity, negatively impacting the quality of life [3,6]. To date, there is no antiviral therapy for TBE, treatment is supportive [3].

Tick-borne encephalitis has a low notification rate (< 0.5/100,000 population at country level) in the European countries of the Mediterranean basin [1]. Nonetheless, recent evidence indicates an increasing risk of TBE in south-western Europe [7], including Italy [8-11].

According to the Italian legislation, TBE has been mandatorily notifiable to the National Public Health Institute (Istituto Superiore di Sanità (ISS)) since the establishment of the national surveillance system for TBE in 2017 [12]. The highest notified TBE incidences, including settings of high endemicity, are in the forested and mountainous Alpine environments in the north-eastern part of the country, referred to as Triveneto [13]. This area comprises the autonomous provinces (APs) of Trento and Bolzano and the regions of Veneto (seven provinces) and Friuli-Venezia Giulia (four provinces).

Italy has a decentralised national healthcare system where regional/AP governments are responsible for prevention and response to vector-borne diseases. Prevention of TBE in humans relies predominantly on voluntary vaccination of humans and secondarily on personal protection measures (wearing protective clothing, using vector repellents) and milk pasteurisation [12]. Immunisation programmes against TBE were introduced in Triveneto regions/APs between 2000 and 2010, with vaccination currently provided free of charge to all residents or persons with occupational exposure in this area [14,15]. However, vaccination coverage is low: < 6% for one or more vaccine doses in Friuli-Venezia Giulia in 2016 [16] and < 10% for a 3-dose primary vaccine scheme in Trento AP in 2017–2024 [17].

Determining the burden of TBEV infections using composite health measures is crucial for disease prioritisation, risk assessments and prevention policies [18]. To improve understanding of the impact of TBEV infections in Triveneto and support public health decision-making, we estimated the burden of TBEV infections by calculating disability-adjusted life years (DALYs) in 2017–2024, overall and by age, sex, year and geographical area.

## Methods

### Study area

We analysed data on notified TBE cases from 2017 to 2024 in Triveneto. In January 2024, the estimated population of Triveneto was 7,105,761 residents (ca 12% of the Italian population). Triveneto borders Austria in the north and Slovenia in the east, featuring a mountainous and largely forested landscape with presence of TBEV reservoir hosts (mainly small rodent species) and records of TBEV infections [13,19]. The APs of Trento and Bolzano and the province of Belluno in Veneto region are predominantly mountainous [20] whereas the southern areas cover the eastern part of the Po valley and are considered low risk areas for TBEV infection [13].

### Case definitions

Notification of TBE in Italy is based on the European Union (EU) standardised case definition [21]. Cases are categorised as probable or confirmed according to the criteria outlined (Box) and as autochthonous or imported based on travel history and most probable area of exposure, considering the incubation period for TBE (7 days on average, up to 28 days; 4 days for food-borne infection) and the travel destination [12].

Box **Definitions of probable and confirmed cases of tick-borne encephalitis, Italy^a^**

**Probable case:**

• Any person in Italy with signs of inflammation of the central nervous system (meningitis, meningoencephalitis, encephalomyelitis, encephaloradiculitis)

AND

• Detection of TBEV-specific IgM antibodies in a single serum sample

OR

• Epidemiological link: exposure to the same unpasteurised milk product as a confirmed case or possible exposure to a tick bite in an endemic area or residence in an endemic area.

**Confirmed case:**

• Clinical criteria as for probable case

AND

• Detection of TBEV with PCR in a blood and/or cerebrospinal fluid (CSF) sample or isolation of TBEV from a clinical sample or TBEV-specific IgG and IgM antibodies in a serum or CSF sample or seroconversion of TBE-specific antibodies in a paired serum sample.

TBEV: tick-borne encephalitis virus.^a^ Based on European Union case definitions [21].

### Outcome measure and disease model

**Figure 1:**
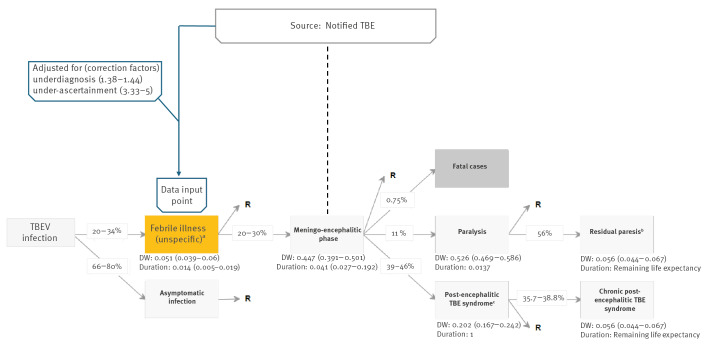
Tick-borne encephalitis virus infection outcome tree and infection/disease specific parameters, Triveneto, north-east Italy, 2017–2024

DW: disability weight; R: recovered; TBEV: tick-borne encephalitis virus.

We used DALYs as a measure of burden of disease. Disability-adjusted life years are a composite time-based measure which combines years of life lost due to premature death (YLLs) and years of healthy life lost due to disability (YLDs) [22]. We estimated the burden per calendar year and the mean annual number of YLDs, YLLs, DALYs and DALYs per 100,000 population, overall and stratified by sex, age and geographical area at level 2 of the nomenclature of territorial units for statistics (NUTS2) and level 3 (NUTS3) [23]. We used the Burden of Communicable Disease in Europe (BCoDE) toolkit developed by the European Centre for Disease Prevention and Control (ECDC) [24]. The underlying methodology, transition probabilities of clinical progression, disability weights and disability duration per health outcome of the BCoDE-TBE disease model have been previously described [25]. In all models, we applied fixed disability weights and transition probabilities across the different age groups. Presentation of the TBE disease model outcome tree and parameters used are described in Figure 1.

The duration of a condition was estimated in years. The figure has been adapted from [24].

^a^ Febrile illness (unspecific) refers to persons who experience symptoms such as headache, malaise, myalgia, anorexia, nausea, and/or vomiting (a subset of these cases progress to the meningo-encephalitic phase), attributed to TBEV infection.

^b^ Lifelong residual paralysis corresponds to a health state with partial loss of or impaired movement attributed to mild motor impairments.

^c^ Post-encephalitic TBE syndrome refers to a health state with symptoms including cognitive or neuropsychiatric complaints, balance disorders, headache, dysphasia, hearing defects and spinal paralysis.

### Model input data and computational analysis

The BCoDE tool requires incidence data of unspecific febrile illness attributed to TBEV infection for the DALY estimation. In our analysis, the annual number of notified confirmed autochthonous TBE cases with probable infection, exposure and/or residence in Triveneto represented the raw input to the TBE outcome tree model. These data were complemented with estimations on underdiagnosis of TBE (i.e. persons with TBE diagnosed as unspecified arthropod-borne viral encephalitis) [26,27] and under-ascertainment of non-neuroinvasive symptomatic TBEV infection (i.e. persons with mild TBEV infections not seeking healthcare), derived from the BCoDE-TBE model transitional probabilities [24,28] (Figure 1). Then, through built-in functions in the tool, we could calculate the incidence of TBEV infections presenting febrile illness, a subset of which will progress to cases with neuroinvasive manifestations. Details on the underestimation factor estimations and related terminology definitions are provided in Supplementary Note 1.

Separate runs of the BCoDE tool, with corresponding model inputs and adjustments, were applied across the study area: the entire Triveneto, the regions and autonomous provinces (NUTS2), and provinces within the Triveneto regions (NUTS3). We used a multinomial logistic regression model to impute the NUTS3 area of probable infection/exposure or residence for all notified infections missing this information (i.e. 3.4% of all notified TBE), based on the distributions of observed NUTS2 and NUTS3 areas of exposure/residence. Details on the allocation of TBEV infections per region/AP and province are provided in Supplementary Note 2.

Input datasets were stratified by 19 age groups (< 1 years, 1–4 years, followed by age groups of 5 years and a final group ≥ 85 years) and where applicable, by sex (male/female). A subset of the datasets was stratified by 10-year age groups to avoid overestimating statistical differences across groups. Corresponding age group weighted life-expectancy estimations were calculated using Triveneto population size estimates and the 2010 global burden of disease (GBD) standardised life expectancy table [29] integrated into the BCoDE tool. For population-based estimates, we used the census estimates of the Italian population for 2024, obtained from the Italian National Institute of Statistics (Istat) [30].

Uncertainty intervals (UI) per disease model were expressed using uniform or PERT (program evaluation and review technique) input parameter distributions (i.e. for underdiagnosis and under-ascertainment, health outcome transition probabilities, health outcome duration and health outcome disability weights). Using the BCoDE application, we run Monte Carlo simulations of the TBE progression model parameters with 10,000 iterations, without time discounting and age-weighting. We used the resulting median DALYs and their 95% UIs to compare groups of interest [28]. We performed two sensitivity analyses, altering the transition probabilities and associated parameters between the health states: febrile illness (unspecific) — meningo-encephalitic phase and meningo-encephalitic phase — paralysis, as described in detail in Supplementary Note 3, to assess uncertainty around the estimated burden attributed to TBE.

## Results

From 2017 to 2024, 295 confirmed autochthonous TBE cases were notified in Triveneto. Two cases with a missing date of birth were excluded from the analysis. For the remaining cases, the mean number of notified cases per year was 36.4 (range: 16–67). Median age at symptom onset was 56 years (range: 0–91 years). Of the 293 cases, 67% (n = 197) were males and 33% (n = 96) females.

After adjusting for under-ascertainment and underdiagnosis, we estimated an annual mean of 209 (95% UI: 203–217) symptomatic TBEV infections. These infections resulted in 41.18 (95% UI: 38.74–43.78) DALYs per year, corresponding to an annual 0.20 (95% UI: 0.19–0.21) DALYs per case and 0.58 (95% UI: 0.55–0.62) DALYs per 100,000 population per year (Table). Years of healthy life lost due to disability contributed 67.3% of the annual burden and YLL the remaining 32.7%. Late sequelae (post-meningo-encephalitic phase) accounted for 63.3% of the estimated DALYs.

**Table:** Mean annual disease burden estimates of symptomatic tick-borne encephalitis virus infections, by region/autonomous province, Triveneto, north-east Italy, 2017–2024^a^

Region or autonomous province	YLD	95% UI	YLL	95% UI	DALYs	95% UI	DALYs/100,000	95% UI
Bolzano	1.65	1.51–1.80	0.80	0.74–0.87	2.45	2.25–2.67	0.46	0.42–0.50
Trento	10.39	9.66–11.19	5.02	4.68–5.37	15.41	14.35–16.53	2.84	2.65–3.05
Veneto	12.57	11.78–13.36	6.11	5.78–6.47	18.69	17.59–19.80	0.39	0.36–0.41
Friuli-Venezia Giulia	3.10	2.86–3.36	1.50	1.40–1.62	4.61	4.27–4.97	0.39	0.36–0.42
Total	27.73	26.00–29.59	13.45	12.67–14.25	41.18	38.74–43.78	0.58	0.55–0.62

DALYs: disability-adjusted life years; UI: uncertainty interval; YLD: years of healthy life lost due to disability; YLL: years of life lost due to premature death.

^a^ Neuroinvasive and non-neuroinvasive symptomatic infections were included.

**Figure 2:**
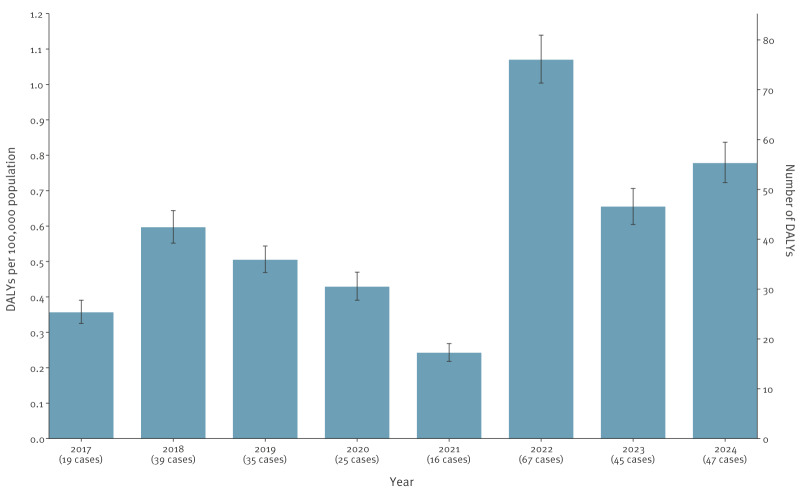
Estimated annual burden of symptomatic infections with tick-borne encephalitis virus, Triveneto, north-east Italy, 2017–2024

The TBEV infection burden peaked in 2022 with 1.07 (95% UI: 1.00–1.14) DALYs per 100,000 population, corresponding to a 3.0- and 4.4-fold burden increase compared with 2017 and 2021, respectively (the years with the lowest TBE burden) (Figure 2).

DALYs: disability-adjusted life years.

Error bars correspond to 95% uncertainty intervals. The number of notified TBE cases is provided in the label of the X-axis.

**Figure 3:**
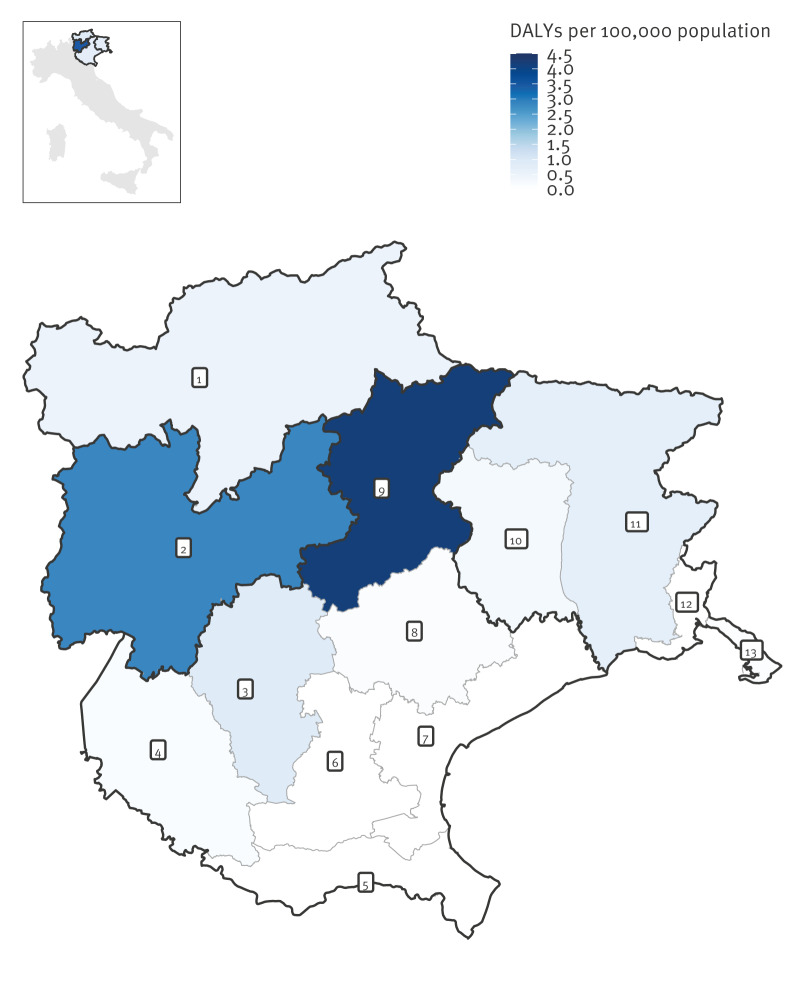
Estimated mean annual burden of symptomatic infections with tick-borne encephalitis virus, Triveneto, north-east Italy, 2017–2024

DALYs: disability-adjusted life years; NUTS: nomenclature of territorial units for statistics level 2 (NUTS2) and level 3 (NUTS3).

Geographically, at region/AP level (NUTS2), the estimates were highest for the AP of Trento with 2.84 (95% UI: 2.65–3.05) DALYs per 100,000 population per year. For the other regions/APs, the estimates were below 0.65 DALYs per 100,000 population per year (Table). However, the Veneto region alone accounted for ca 45% of the total burden. At province level (NUTS3), TBE burden was highest in Belluno (Veneto region) with 4.17 (95% UI: 3.88–4.46) DALYs per 100,000 population per year, followed by Vicenza (Veneto region) and Udine (Friuli-Venezia Giulia region) with < 1 DALY per 100,000 population per year (Figure 3). The lowest estimates were for the provinces of Rovigo, Padova, Venezia (Veneto region) and Gorizia (Friuli-Venezia Giulia region).

The geographical areas were numbered: 1: Bolzano autonomous province (533,267 residents) (NUTS2); 2: Trento autonomous province (542,050 residents) (NUTS2); Veneto region provinces (NUTS3; 4,838,253 residents): 3: Vicenza; 4: Verona; 5: Rovigo; 6: Padova; 7: Venezia; 8: Treviso; 9: Belluno; Friuli-Venezia Giulia region provinces (NUTS3; 1,192,191 residents): 10. Pordenone; 11: Udine; 12: Gorizia; 13: Trieste.

The point estimates and their corresponding 95% uncertainty intervals are provided in Supplementary Table 1.

**Figure 4:**
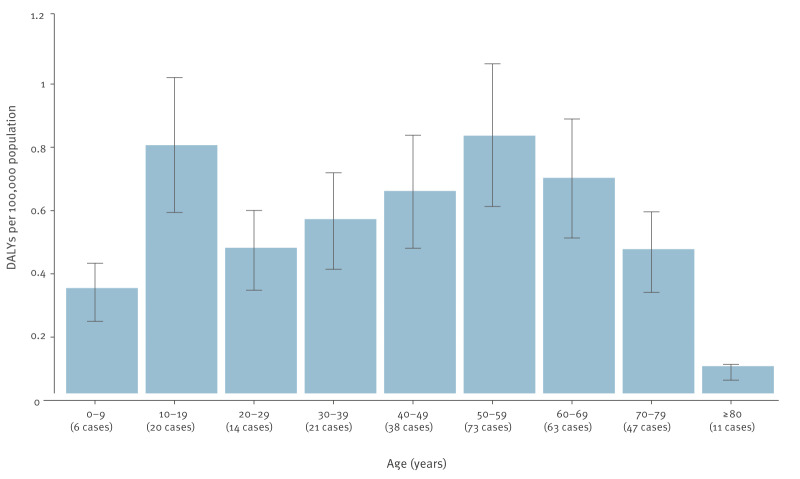
Estimated mean annual burden of symptomatic infections with tick-borne encephalitis virus, by age, Triveneto, north-east Italy, 2017–2024

Overall, males accounted for 67.1% of the mean annual DALYs corresponding to 0.79 (95% UI: 0.73–0.85) DALYs per 100,000 population. The estimate for females was 0.38 (95% UI: 0.34–0.41) DALYs per 100,000 population per year. Across age groups, the annual point estimates were  < 1 DALYs per 100,000 population of the respective age group, peaking in the age groups of 50–59 years and 10–19 years with 0.81 (95% UI: 0.61–1.06) and 0.78 (95% UI: 0.59–1.02) DALYs per 100,000, respectively (Figure 4). The sex-age stratified estimates were highest in males aged 30–39, 40–49, 50–59 and 60–69 years. More details of the DALYs per 100,000 population and their corresponding 95% UIs stratified by age and sex are provided in Supplementary Figure 1.

DALYs: disability-adjusted life years.

Error bars correspond to 95% uncertainty intervals. The numbers of notified TBE cases are provided in the label of the X-axis.

As presented in Supplementary Note 3, the sensitivity analyses of population-based burden estimates did not significantly differ from the main analysis results, however, the DALYs per case per year estimates were ca threefold that of the main analysis.

## Discussion

In this study, we assessed the years of full health lost due to TBEV infections in Triveneto, north-east Italy, where the disease is endemic. We estimated an annual burden of 0.58 (95% UI: 0.55–0.62) DALYs per 100,000 population in 2017–2024 and observed geographical and temporal differences.

Our results of DALYs for Triveneto are comparable to the TBE burden estimated for the EU/EEA for 2009–2013 (0.69; 95% UI: 0.65–0.74 DALYs/100,000/year) [28]. However, our estimates for the most affected province, Belluno, were lower than the mean annual burden (10.95; 95% UI: 10.25-11.65 DALYS/100,000/year) estimated for 2009–2013 in Slovenia, which has one of the highest TBE notification rates in Europe [25].

In 2022–2024, TBEV infections had an elevated impact on Triveneto’s population health, reflecting the higher number of TBE cases notified in this period [11]. Awareness among healthcare professionals and more frequent testing could have contributed to the increase in notified cases, however, these increases align with climatic anomalies in the same years likely favouring tick abundance and TBEV transmission [7,19,31-33].

Geographically, the highest impact of TBEV infections was seen in the AP of Trento and Belluno province, indicating these as priority areas for TBE prevention. These findings are in line with other studies showing that these areas are suitable for TBEV transmission [19,34,35]. Conversely, no burden of TBEV infections was observed in several neighbouring provinces (e.g. Rovigo and Venezia), likely attributed to local geomorphological and land cover characteristics, including low altitude and low forest coverage, both of which are negatively associated with tick abundance and TBEV infection risk [13,19].

Considering the high effectiveness of licenced TBE vaccines (> 92%) against infection with the European TBEV subtype [36], our findings likely reflect persisting vaccination coverage gaps in the AP of Trento and in Belluno province, despite increasing numbers of TBE vaccination in Trento in recent years (increasing from 16,525 vaccine dose administrations in 2018 to 42,936 in 2023) [17]. Specifically, people with a high risk of occupational exposure (such as farmers, hunters, forest workers) may not be optimally vaccinated, as indicated in a study from Trento where < 25% of a convenience sample of agricultural workers were vaccinated against TBE in 2017 [37].

As the transmission pattern of TBEV is focal in north-east Italy [13], robust assessment of high risk TBEV infection areas across Triveneto, coupled with analyses of vaccination coverage may support fine-scale vaccine rollout programmes, thus considerably reducing the TBEV infection burden in Trento, Belluno and other areas of high TBEV transmission.

We observed a similar burden of TBEV infections by age, despite the higher TBE incidence in those aged ≥ 50 years compared with younger persons [10,11]. Although there could be differences in TBE vaccination coverage between age groups (outweighing age-dependent risk), our findings strengthen the importance of TBE vaccination of persons aged > 1 year, especially among Trento and Veneto residents frequently visiting high TBEV risk areas. Vaccination of Italian and foreign tourists of all ages visiting the mountains in Trento and Belluno for hiking, trekking or other outdoor activities should also be considered [38].

In line with previous studies [25], we estimated a higher TBEV infection burden in males compared with females, especially in those aged ≥ 30 years. Awareness of TBE appears to be low among working-age Italian males with outdoor occupations associated with high exposure risk to ticks [37]. Hence, increasing TBE risk perception in these groups may complement vaccination efforts and contribute to a consistently low TBEV infection burden in endemic areas.

Finally, the estimated high burden of TBEV infection attributed to late sequelae (> 63%), indicates the importance of neuroinvasive infection long-term follow-up and supportive care provision to affected persons, in view of alleviating long-term neurological and neurocognitive impairments attributed to TBE.

Our study has several limitations. Firstly, our results likely underestimate the true burden of TBEV infections in north-east Italy as our analysis did not account for under-notification of diagnosed neuroinvasive TBEV infections. Secondly, the TBE model integrated in the BCoDE tool considers all non-neuroinvasive TBEV infections as mild symptomatic cases. However, recent evidence [39] shows that patients with a non-neuroinvasive symptomatic TBEV infection are also likely to develop severe health conditions requiring hospitalisation. Hence, by default we underestimated the burden attributed to these cases. Thirdly, through applying fixed disability weights and transition probabilities across the different age groups, we may have over- or underestimated the true number of DALYs attributed to TBEV infections as TBE morbidity varies by age while less is known about long-term sequelae in children [33,40]. Fourthly, our results may portray a somewhat biased geographical distribution of the TBEV infection burden in the Triveneto area, attributed to potential variability in TBE laboratory ascertainment across the study regions/APs/provinces. Additionally, by applying the BCoDE tool – TBE model integrated parameters (i.e. estimates of disability weight, health state duration, transition probability) in our study we may have slightly mis-estimated the true TBEV infection burden by not accounting for health/health system factors specific of the Triveneto population. In this aspect, furthering understanding on the proportion of mild symptomatic TBEV infections that progress to neuroinvasive disease will improve the accuracy of future TBEV infection individual/case burden estimates. Finally, in-depth interpretation of our findings was partially limited by the lack of (publicly available) data on TBE vaccination coverage trends and distribution by area, population groups and vaccination status (e.g. complete vs incomplete vaccination cycles) across the Triveneto regions, APs and provinces.

## Conclusion

We estimated a low TBEV infection burden in north-east Italy unevenly distributed between Triveneto APs/provinces. Robust assessment of areas at high risk of TBE may help to plan targeted TBE vaccination programmes aimed at increasing coverage among local residents, with the objective of maintaining TBE as a low-burden disease.

## Data Availability

The datasets presented in this article are not readily available due to Italian data protection legislation. Register data in Italy is accessible for researchers with approval from the relevant authorities. Requests to access the datasets should be directed to Istituto Superiore di Sanità (ISS).

## Members of the Tick-borne encephalitis group with affiliations

Directorate of Prevention, Food Safety, and Veterinary Public Health-Veneto Region, Venice: Filippo da Re, Francesca Zanella, Davide Gentili; Central Directorate for Health, Social Policies, Friuli Venezia Giulia Regione, Udine: Sarah Samez, Cristina Schellenberger; Department of Prevention, Provincial Health Authority, Trento: Chiara Mocellin; Laboratory of Microbiology and Virology, South Tyrol Health Authority: Elisabetta Pagani.

## Supplementary Data

319000 Supplementary Material
