# Supplementary material for: Assessing the annual burden of tick-borne encephalitis virus infections, north-east Italy, 2017 to 2024
Source: Euro Surveill. 2026 Apr 30;31(17):2500733. doi: 10.2807/1560-7917.ES.2026.31.17.2500733 (PMC13135150; doi:10.2807/1560-7917.ES.2026.31.17.2500733)
Supplement: Supplementary Material [file 25-00733_FOTAKIS_Supplement.pdf]

Supplementary file: Methodology details and tick-borne encephalitis virus infection associated DALY estimates stratified by sex, age and Autonomous Province/province

This supplementary material is hosted by *Eurosurveillance* as supporting information alongside the article [Assessing the annual burden of tick-borne encephalitis virus infections, north-east Italy, 2017 to 2024], on behalf of the authors, who remain responsible for the accuracy and appropriateness of the content. The same standards for ethics, copyright, attributions and permissions as for the article apply. Supplements are not edited by *Eurosurveillance* and the journal is not responsible for the maintenance of any links or email addresses provided therein

Supplementary note 1: Study definitions and underestimation factors

Under-diagnosis (of persons with TBE): Can be understood as TBE patients/cases which are diagnosed (in the context of this study) with unspecified arthropod borne viral encephalitis, hence are not captured by the surveillance system.

Under-ascertainment (of non-neuroinvasive symptomatic TBEV infections): Can be understood as cases presenting febrile illness symptoms (that do not develop central nervous system manifestations at a later timepoint), which do not seek healthcare and/or if they do are not reported, and hence are missed by the surveillance system.

Under-notification (of diagnosed TBE cases): Can be understood as TBE cases which are diagnosed as such but are not notified through the notification system hence are not captured by the surveillance system.

Adjusting for under-diagnosis: We applied a range of multipliers in the BCoDE tool: 1.38-1.44, for all age groups. These values were derived from a national scale analysis from Italy (data not shown) showing a 27-31% underdiagnosis rate of persons with TBE in Italy for the years 2017-2023. Specifically, the underdiagnosis rate was estimated by: using the Italian Hospital Discharge Records (HDRs) for the years 2017-2023 and specific diagnostic ICD-9-CM codes; imputing the aetiology of all discharges with a diagnosis of unspecified arthropod borne viral encephalitis through a multinomial logistic regression model; and assessing the number of these cases against the number of TBE cases reported to the national TBE surveillance system. The estimated values are in line with the findings of [1] (from Germany) and [2] (from Slovenia).

Adjusting for under-ascertainment: Following the same approach described in [3] we applied a range of multipliers: 3.33-5, for all age groups, given that according to the toolkit 20-30% of symptomatic cases will progress to the neuroinvasive phase.

Supplementary note 2: Geographical case allocation

For surveillance datasets corresponding to the years 2017-2019 we geographically allocated TBE cases based on their municipality of domicile and, when this was not present, on their municipality of notification. For the 2020-2024 surveillance datasets, the municipality of domicile and notification were not available as discrete variables; therefore, we used the suspected municipality of infection/exposure as identified in the case investigations. In 55% of cases, the suspected municipality of infection/exposure corresponded with the municipality of domicile. When this information was not available, we used the region or Autonomous Province of notification.

Ten notified TBE cases (3.4% of the total) had information on the region of domicile/ exposure/notification (NUTS2) yet lacked province level (NUTS3) geographic information (5 allocated to the region of Veneto and 5 to Friuli-Venezia Giulia). For these cases, we used a multinomial logistic regression model to impute the province (NUTS3) of likely domicile/exposure/notification. Essentially, following a similar approach described in [4] we conducted an iterative form of stochastic imputation (based on the previously identified variables of NUTS2 and NUTS3 area of domicile/exposure/notification), using the distribution of the observed data to estimate missing information (here the missing NUTS3 area of residence/exposure/notification). We run an analysis with 200 imputations of the missing outcome. For each individual case missing NUTS3 level information, we assigned the imputed NUTS3 value with the highest frequency amongst the corresponding imputed outcomes. The estimated outcome was then used in the mapping analysis at province (NUTS 3) level.

Supplementary note 3: Sensitivity analyses

Considering for varying health state transition probabilities reported in the literature, we performed two sensitivity analyses to assess uncertainty around the estimated burden attributed to TBEV infections. In sensitivity analysis 1, we modified the transition probability between "febrile ilness" and "Meningoencephalitic phase" from 20-30% to 80%, and adjusted accordingly for under-ascertainment (i.e. under-ascertainment factor of 1.25). The model in sensitivity analysis 2 was built on the model of sensitivity analysis 1, with the addition of a reduction in the transition probability between "Meningoencephalitic phase" and "Paralysis" from 11% to 6%.

Sensitivity analysis 1 results (for symptomatic TBEV infections in the Triveneto area): annual burden of 40.47 (95% UI: 39.73-41.23) DALYs, corresponding to 0.63 (95% UI: 0.62-0.64) DALYs per case per year, and 0.57 (95% UI: 0.56-0.58) DALYs per 100,000 population per year.

Sensitivity analysis 2 results (for symptomatic TBEV infections in the Triveneto area): annual burden of 37.69 (95% UI: 36.96-38.44) DALYs, corresponding to 0.58 (95% UI: 0.57-0.60) DALYs per case per year, and 0.53 (95% UI: 0.52-0.54) DALYs per 100,000 population per year.

Supplementary Table 1: Estimated mean annual disability-adjusted life years per 100,000 population due to tick-borne encephalitis virus infections, by Autonomous Province/province, Triveneto (North-East Italy), 2017–2024

**Autonomous Province /Province**	**DALYs/100,000 pop (95% UI)**
Bolzano	0.46 (0.42-0.50)
Trento	2.84 (2.65-3.05)
Verona	0.18 (0.16-0.20)
Vicenza	0.80 (0.86-0.92)
Belluno	4.17 (3.88-4.46)
Treviso	0.16 (0.15-0.18)
Venezia	0
Padova	0
Rovigo	0
Udine	0.66 (0.61-0.71)
Gorizia	0

Trieste	0.13 (0.11-0.17)
Pordenone	0.29 (0.24-0.35)

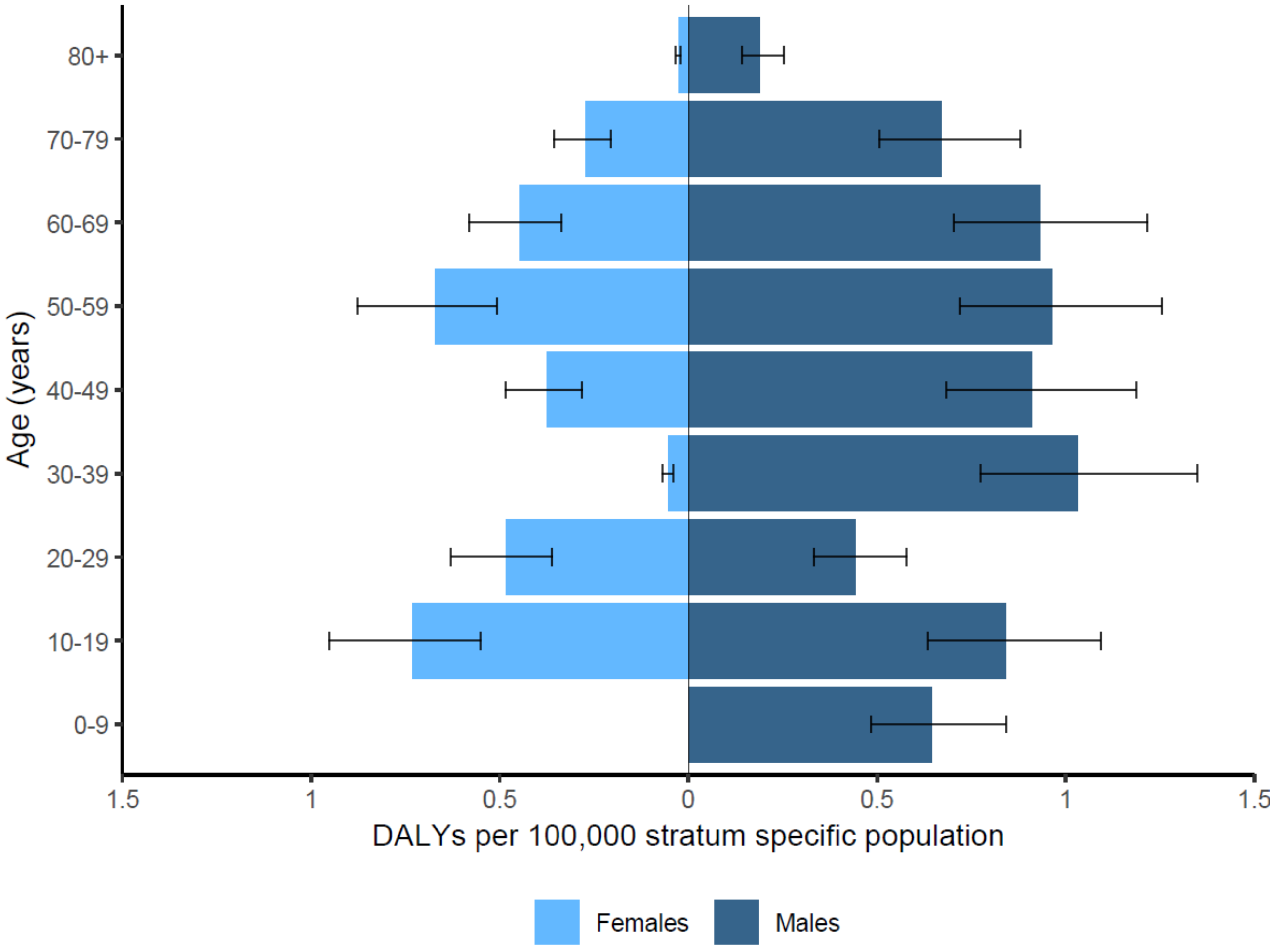

Supplementary Figure 1: Estimated mean annual disability-adjusted life years per 100,000 stratum-specific population due to tick-borne encephalitis virus infections, by age and sex, Triveneto (North-East Italy), 2017–2024.

References

1. Schley K, Friedrich J, Pilz A, Huang L, Balkaran BL, Maculaitis MC, et al. Evaluation of under-testing and under-diagnosis of tick-borne encephalitis in Germany. BMC Infect Dis. 2023 Mar 7;23(1):139.

2. Šmit R, Postma MJ. The Burden of Tick-Borne Encephalitis in Disability-Adjusted Life Years (DALYs) for Slovenia. Munderloh UG, editor. PLOS ONE. 2015 Dec 16;10(12):e0144988.

3. Cassini A, Colzani E, Pini A, Mangen MJJ, Plass D, McDonald SA, et al. Impact of infectious diseases on population health using incidence-based disability-adjusted life years (DALYs): results from the Burden of Communicable Diseases in Europe study, European Union and European Economic Area countries, 2009 to 2013. Eurosurveillance [Internet]. 2018 Apr 19 [cited 2025 May 20];23(16). Available from: https://www.eurosurveillance.org/content/10.2807/1560-7917.ES.2018.23.16.17-00454

4. Pezzotti P, Bellino S, Riccardo F, Lucaroni F, Cerquetti M, Pantosti A, et al. Vaccine preventable invasive bacterial diseases in Italy: A comparison between the national surveillance system and recorded hospitalizations, 2007–2016. Vaccine. 2019 Jan;37(1):41–8.
